# Carotid bruits as predictor for carotid stenoses detected by ultrasonography: an observational study

**DOI:** 10.1186/1471-2377-8-23

**Published:** 2008-06-24

**Authors:** Elias P Johansson, Per Wester

**Affiliations:** 1 Department of public health and clinical medicine, medicine, Umeå, Sweden

## Abstract

**Background:**

Carotid surgery in asymptomatic subjects with carotid stenosis is effective to prevent ischemic stroke. There is, however, uncertainty how to find such persons at risk, because mass screening with carotid artery ultrasonography (US) is not cost-effective. Signs of carotid bruits corresponding to the carotid arteries may serve as a tool to select subjects for further investigation. This study is thus aimed at determining the usefulness of carotid bruits in the screening of carotid stenoses.

**Methods:**

1555 consecutive carotid ultrasonography investigations from 1486 cases done between January 2004 and March 2006 at Norrlands University Hospital, Sweden, were examined. 356 subjects, medium age 69 (27–88) years, had a significant (≥ 50%) US-verified carotid stenosis uni- or bilaterally, 291 had been examined for signs of carotid bruits. The likelihood ratios for carotid bruits to predict US-verified carotid stenoses were calculated and expressed as likelihood percentages.

**Results:**

Thirty-one out of 100 persons (31%) with carotid bruit as an indication to perform carotid US had a significant (≥ 50%) carotid stenosis. 281 of the 356 (79%) cases with significant carotid stenoses were found among patients with cerebrovascular disease (CVD). 145 of 226 (64%) CVD patients with a significant carotid stenosis had a carotid bruit. In patients with 50–99% carotid stenoses carotid bruits had an accuracy of 75% (436/582), a sensitivity of 71% (236/334), a specificity of 81% (200/248), a positive likelihood ratio at 3.65 and a negative likelihood at 0.36. Patients with 70–99% stenoses had the highest sensitivity at 77% (183/238). In patients with 100% carotid stenoses, carotid bruits had a sensitivity of 26% (15/57) and a specificity of 49% (256/525).

**Conclusion:**

Although carotid bruits are not accurate to confirm or to exclude significant carotid stenoses, these signs are appropriate for directed screening for further investigation with carotid US if the patient lacks contraindications for surgery. Lack of carotid bruits in CVD patients does not exclude a carotid stenosis.

## Background

Carotid endarterectomy (CEA) in patients with asymptomatic carotid stenoses aims at reducing the risk of stroke. There has previously been controversies about the risk and benefit ratio of CEA in asymptomatic cases[1]. An asymptomatic stenosis was previously often considered to be a negative prognostic factor that was not significantly altered by surgical interventions [2-4]. The asymptomatic carotid stenosis trial (ACST)[5] published in 2004 more than doubled the number of patients studied in randomized clinical trials on this indication. The study showed a clear-cut beneficial effect, albeit not as large as CEA in symptomatic patients with carotid stenosis. The ACST and the subsequent Cochrane meta-analysis[6] led to a policy shift in many countries, including Sweden, to offer CEA among asymptomatic individuals with significant carotid stenosis. It is therefore important to find eligible asymptomatic individuals with carotid stenoses where CEA or carotid endovascular treatment may be appropriate [5,6].

It has been estimated that it is cost effective to screen a population with carotid ultrasonography (US) if the prevalence exceeds 4.5%[7]. However the U.S. Preventive Services Task Force concluded that a prevalence of 5% would render a large number of false positive carotid stenoses since carotid US is not 100% specific, leading to that the gain of CEA for these individuals were reduced [8]. However this problem is reduced if the prevalence in the screened population is higher than 5%. In the unselected general population, the prevalence is substantially lower than 5%. Therefore, a sign of a carotid bruit might be used as an indication for carotid US in order to detect a carotid stenosis. Thus, knowledge of the accuracy of signs of carotid bruits may be important and so is the knowledge about what type of stenoses that produces carotid bruits. Many studies have been conducted on this topic [9-12] with very different results. An ongoing study investigates the usage of the specialized Markov ultrasound instrument as a method of mass screening of older patients[13] as another method of finding asymptomatic carotid stenoses. The American Society of Neuroimaging guidelines for screening of extracranial carotid artery stenosis states that screening of the general population is not recommended but on subpopulations aged 65 years and above with at least three risk factors (hypertension, coronary artery disease, smoking or hyperlipidemia) it needs to be considered[14].

To ensure a high number of yearly CEA operations per surgeon, all patients from the northern part of Sweden (i.e., from the counties of Norrbotten, Västerbotten, Västernorrland and Jämtland with a combined population of 880 000[15]) are referred to Umeå Stroke Center at Norrlands University Hospital for a detailed clinical, carotid US and radiological examination, information and decision about CEA.

The overall aim of this study is to examine carotid bruits; if a carotid bruits as an indication for carotid US yields a high, cost-effective, percentage of significant carotid stenoses (i.e., around 10% or higher), how this compares to symptomatic cerebrovascular disease (CVD) and other indications for carotid US and to calculate statistical data on how effective carotid bruits are to predict a significant carotid stenosis with carotid US as the gold standard. In addition, this manuscript also aims to introduce likelihood percentages as a complement to likelihood ratios.

## Methods

This report is based on observational data collected as an assurance of quality of health care. Thus, no approval from the local ethics committee was sought.

A search was made for all Ultrasound Sonography (US) examinations of the carotid arteries recorded in "the patient administrative system, PAF," at the Department of Clinical Physiology at Norrlands University Hospital, Sweden, between 1 January 2004 and 31 March 2006. All carotid US investigations at Norrlands University Hospital were conducted at the Department of Clinical Physiology. All the US examinations were performed with an Acuson Sequia 512; Siemens Medical, Munich, Germany. The peak systolic velocity in the common carotid artery (CCA) and internal carotid artery (ICA) were recalculated into a degree of stenosis in those arteries according to the CCA criteria[16], which have been validated locally against the NASCET criteria for angiography[17]. As a clinical routine, all patients who are referred from secondary or tertiary hospitals within the northern region of Sweden to Norrlands University Hospital for carotid surgery investigation are re-examined at the Department of Clinical Physiology. 1555 carotid US examinations in 1486 patients were done during this period.

**Figure 1:**
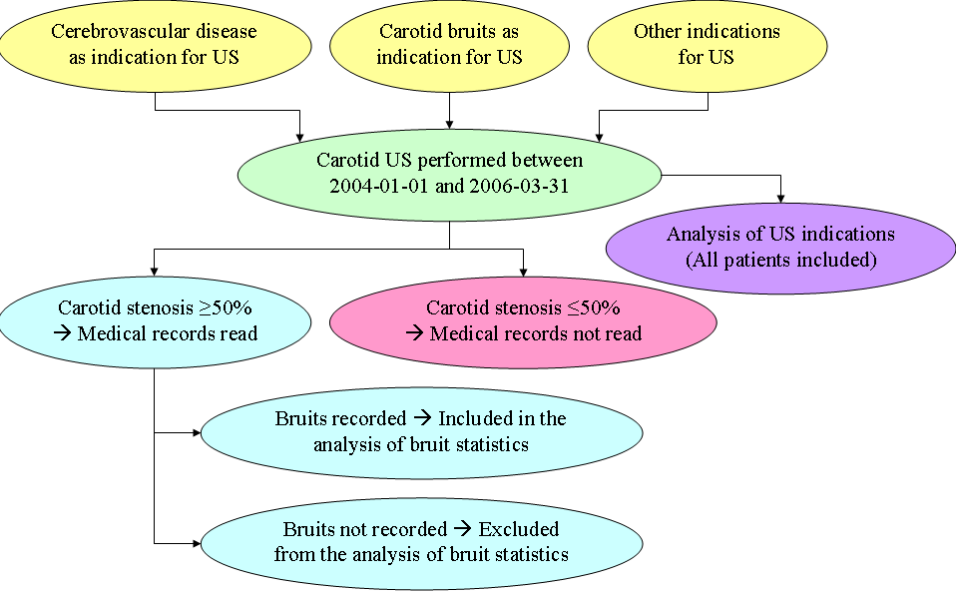
**Schematic algorithm over the inclusion and exclusion of patients**. All patients that underwent a carotid ultrasonographic (US) examination between 2004-01-01 and 2006-03-31 where included in the analysis of US indications. The patients with a carotid stenosis ≥ 50% and the presence or absence of a carotid bruit in their medical records were included in the analysis of carotid bruit statistics.

All 1555 US examinations referrals and answers were examined according to a pre-specified protocol. This protocol included the patients' age, gender, indication for the carotid US examination, the degree of stenosis of the common carotid artery (CCA) or the internal carotid artery (ICA) on each side and the presence of blocking echo shadows. The number of US examinations made on each indication was compared with the number of found stenoses. 1158 of the 1555 carotid US examinations did not yield any sign of a carotid stenosis and were not further studied. 35 examinations from 33 patients were excluded because they were the second or third time the patient was examined, rendering only one examination per patient. Five patients were excluded because of missing medical records and one subject because the record was classified. In total 356 examinations performed on 356 patients were included; their medical records were read in detail. A schematic algorithm of the inclusion and exclusion of patients is presented in figure 1.

The grade of stenosis was divided into four categories: 0–49% – No significant stenosis; 50–69% – Low grade stenosis; 70–99% – High grade stenosis; and 100% – occlusion. A 50–99% combination group was also analyzed. The US examination does not allow for accurate determination of stenoses below 50%. Moreover, 70% is the current cut-off for asymptomatic surgery at Norrlands University Hospital, based on the ACST-study[5,16].

In addition to the previous internal validation, the carotid US was compared with carotid angiographic investigations as a gold standard in 87 patients. 15 cervical sides could not be compared because of echo shadow on the US. The remaining 159 cervical sides were analyzed independently in each patient. 80% (70/87) of the patients had done a computer tomography angiography, 9% (8/87) a conventional angiography, 2% (2/87) a MRI angiography and 8% (7/87) a combination of the three methods. Because all types of angiographies would only be compared with the carotid US, no distinction was made between the different angiographic methods. The overall accuracy between a significant (≥ 50%) carotid stenosis on carotid US and carotid angiography was 83% (132/159). The positive likelihood ratio (PLR) of 50–99% stenoses was 10.2. PLR was above 10 in all categories except the low grade stenoses. The negative likelihood ratio (NLR) was below 0.1 among patients with carotid occlusion. All the specificities were higher than the sensitivities. Carotid US was deemed validated against angiography.

The carotid bruits were divided into "Yes" and "No". Statistical data were calculated for the carotid bruits with the carotid US examination as a gold standard. In 18% (65/356) of the cases the presence or absence of a carotid bruit were not registered in the medical record; these cases were excluded from this analysis. The remaining 291 patients (582 cervical sides) with the presence or absence of a carotid bruit recorded in their medical records were analyzed. Each of the cervical sides was analyzed independently in each patient. In this analysis no consideration was made to whether or not the patients' indication for carotid US was a carotid bruit.

The likelihood ratios have a range from 0 to infinite and are in inverted relations to each other. When these ratios are used as cut-off markers, a positive likelihood ratio above 10 and a negative likelihood ratio below 0.1 are often meaningful. But comparing different likelihood ratios can be difficult because they are inverted and the fact that the difference between, e.g., 2 and 5 is greater than the difference between 10 and 20. In order to facilitate comparisons of different likelihood ratios a system to recalculate the positive and negative likelihood ratios into positive and negative likelihood percentages (PLP and NLP) was created and is presented and discussed in the appendix.

The difference between the sexes was calculated with a chi-square test, using the SPSS 15.0 software. The calculations of sensitivity, specificity, accuracy, likelihood ratios and likelihood percentages were done with the Microsoft Excel 2002 software.

## Results

Thirty-one of 100 (31%) of patients with a carotid bruit as an indication to perform carotid Ultrasonography (US) had a significant (≥50%) carotid stenosis. 281 of 1176 (24%) of the patients with symptomatically cerebrovascular disease (CVD) as an indication to perform carotid US had a significant (≥ 50%) carotid stenosis. 36% (81/226) of the patients with a significant carotid stenosis and with CVD as indication for the carotid US examination did not have a carotid bruit. For the number of examinations done on different indications and their share of the found carotid stenoses see Table 1.

**Table 1:** Demographic data

	Carotid Ultrasound Examinations	FoundCarotid Stenoses
All US indications; number (part of population)	1555 (100%)	356 (100%)
Female; number (part of population)	695 (45%*)	120 (34%*)
Age; mean (range)	66.1 (5–91) years	69.1 (28–88) years
CVD as indication for US; number (part of population)	1176 (76%)	281 (79%)
Bruits as indication for US; number (part of population)	100 (6%)	31 (9%)
Other indications for US; number (part of population)	279 (18%)	44 (12%)

The data for all US examinations (patients with multiple examinations appear multiple times) and the patients who were included in the study. *p < 0.001, chi-square test, demonstrating that men more often had a carotid stenosis than women

As seen in Tables 2 and 3, signs of carotid bruits revealed highest sensitivity and positive likelihood values in the 70–99% carotid stenosis group, while considerably lower values were seen in the 50–69% carotid stenosis group. In the carotid occlusion group, the sensitivity was only 26%, whereas the specificity and accuracy were somewhat higher.

**Table 2:** Statistical data for carotid bruits

	**Grade of Carotid US Stenosis**			
**Carotid Bruits (yes/no)**	**50–69%**	**70–99%**	**100%**	**50–99%**

**Sensitivity**	55% (53/96)	77% (183/238)	26% (15/57)	71% (236/334)
**Specificity**	52% (255/486)	71% (243/344)	49% (256/525)	81% (200/248)
**Accuracy**	53% (308/582)	73% (426/582)	47% (271/582)	75% (436/582)

291 patients (582 cervical sides) had data regarding the degree of carotid stenosis seen on carotid US and presence or absence of a carotid bruit recorded in their medical records. With the US examination as gold standard the sensitivity, specificity and accuracy for carotid bruits were calculated with the hypothesis that all separate stenosis groups are the only ones that produce a bruit in each case

**Table 3:** Statistical data for carotid bruits

	**Grade of Carotid US Stenosis**			
**Carotid Bruits (yes/no)**	**50–69%**	**70–99%**	**100%**	**50–99%**

**Positive Likelihood Ratio**	1.16	2.62	0.51	3.65
**Negative Likelihood Ratio**	0.85	0.33	1.51	0.36
**Positive Likelihood Percent**	14%	62%	-95%	73%
**Negative Likelihood Percent**	15%	67%	-51%	64%

291 patients (582 cervical sides) had data regarding the degree of carotid stenosis seen on carotid US and presence or absence of a carotid bruit recorded in their medical records. With the US examination as gold standard the likelihood ratios and the likelihood percentages for carotid bruits were calculated with the hypothesis that all separate stenosis groups are the only ones that produce a bruit in each case. How to calculate likelihood percentages is presented in the appendix.

## Discussion

The main finding in this study is that a carotid bruit as an indication to perform an US examination gives such a high yield to find a significant carotid stenosis that it is cost-effective[7]. High grade carotid stenoses, 70–99%, are the most likely to produce a carotid bruit whereas a lack of carotid bruit does not exclude a significant carotid stenosis.

The implications from data in this study are that a carotid bruit is a good method for screening large populations during routine auscultation when followed up with a carotid US examination. It is therefore possible to identify subjects with a high risk of asymptomatic stenoses before they become symptomatic, because the prevalence of carotid stenoses in the general population is quite low. For the subpopulation of patients with carotid bruit as US indication, the prevalence of carotid stenoses was higher than 4.5%. Thus it is cost-effective to screen all patients with carotid bruits and CVD with carotid US[7]. Since the prevalence in this sub-population was substantially higher than 5% it probable that the problem with false positive carotid stenoses is reduced[8]. Carotid bruits had a similar yield in identifying patients with significant stenoses as the more common US indication of symptomatic CVD. Hill and colleagues found that bruit and a known history of carotid disease were the only indications that were statistically related to severe carotid disease [18]. The lack of carotid bruits is not accurate enough to exclude CVD patients from an US examination because more than a third of the symptomatic patients with a significant carotid stenosis did not have a carotid bruit. The number of carotid stenoses found in patients with CVD as an indication for their carotid US examination was higher than all the other indications combined. Thus, we conclude that CVD as an US indication is a good way of identifying populations with carotid stenoses, since these stenoses are symptomatic the gain of CEA is larger than the gain of asymptomatic CEA[5,19]. In addition to carotid bruits and CVD, a panel of risk factors (hypertension, coronary artery disease, smoking or hyperlipidemia) can be used as an indication for carotid US in patients above 65 years of age without CVD[14]. Because only the medical records of the patients with a carotid stenosis ≥ 50% were read, the present study cannot verify or falsify this conclusion by the American Society of Neuroimaging[14].

The highest likelihood percentage (PLP) value, specificity and accuracy were found among patients with 50–99% carotid stenoses. It is important to correctly interpret the sensitivity and specificity in the subgroups of carotid stenoses, where the emphasis should be on the sensitivity. The highest sensitivity for the carotid bruits to predict carotid stenoses was found in the 70–99% group, which represents the group of patients with highest risk of stroke. None of the carotid bruit groups met the cut-off value of PLP or NLP > 90%, which is equal to PLR < 10 and NLR < 0.1. Thus, the presence or absence of a carotid bruit is not accurate enough to predict such a diagnosis. Ghilardi did a mass screening of a small community including 16379 individuals in the 45–75 years age span and found that the sensitivity and specificity were 36% and 98% respectively[9] which corresponds to a PLP value of 94% and a NLP value of 35%. Because Ghilardi found a PLP value above 90% they concluded that the presence of a bruit was a good diagnostic marker. Our figures were lower. In the study of Ghilardi, carotid kinking and occlusion were regarded as a carotid lesion[9]. Floriani et al. found a sensitivity and specificity of carotid bruits to predict a carotid lesion of 84% and 40%, respectively[10], rendering a positive likelihood percentage (PLP) value of 29% and a negative likelihood percentage (NLP) value of 60%. Sauve et al. found a sensitivity, specificity, positive and negative likelihood percentages of 63%, 61%, 38% and 39%, respectively, for 70–99% stenoses when studying the NASCET-population[11]. Magyar et al. studied 145 patients of whom 16 had a carotid stenosis of 70–99% and found a sensitivity of 56% and a specificity 91% rendering a PLP value of 84% and a NLP value of 52% for 70–99% stenoses[12] The different definitions of carotid lesions together with different study populations may at least partly explain the discrepancy in findings.

Of all patients examined with carotid US, men had a carotid stenosis in a significantly higher proportion than women. Of the patients included in the study the men to women ratio was 2:1. A similar ratio was seen in the NASCET, ECST and ACST studies[5,19].

There are several limitations of the present study and the most obvious is its non-prospective design. No consideration was made to thorax bruits, and it is possible that some of the carotid bruits recorded in the study originated from, for example, an aortic stenosis; however, this is studied as part of an ongoing prospective study at Norrlands University Hospital (NCT00514592). Some of the carotid bruits among patients with CVD were recorded by physicians just prior to the carotid endarterectomy; these investigators were not unaware of the carotid US findings. However, in some cases no carotid bruit was heard at the CEA admission, which led to another US examination that sometimes revealed that the carotid artery had occluded. The yield of CVD as US indication is not representative among patients with CVD because 188 of the 281 stenoses found were in patients who were referred to Norrlands University Hospital after a stenosis had been suspected at their local hospital. Some patients who were referred had no significant stenosis and were excluded from the study. The yield of symptomatic CVD as an US indication to find a significant carotid stenosis is lower, ≅10–12%, for the patients who were examined for the first time ("the true yield") than the 24% presented in the results section.

## Conclusion

Although signs of carotid bruits are not accurate to confirm or to exclude significant carotid stenoses, the signs of carotid bruits are appropriate for directed screening for further investigation with carotid US if the patient otherwise is suitable for surgery. Symptomatic patients without the presence of a carotid bruit should undergo an US investigation anyway.

## Appendix

In the appendix, we introduce the system of likelihood percentages. Some examples are taken from the results of this study. In the main discussion, Likelihood percentage values are used when different studies' findings of the diagnostic performance of carotid bruits to predict a carotid stenosis are discussed.

Sensitivity is the part of the unhealthy population that has a positive finding in a diagnostic test. Specificity is the part of the healthy population that has a negative finding in a diagnostic test. Predictive values express the combined effect of the sensitivity and the specificity and takes prevalence into account. Likelihood ratios express the combined effect of the sensitivity and the specificity but do not take prevalence into account. Cut-off values in diagnostic tests with a Positive Likelihood Ratio (PLR) > 10 and Negative Likelihood Ratio (NLR) < 0.1 are regarded as significant [20]. By multiplying the prevalence (expressed as odds, pre-test odds) with the likelihood ratio results in the corresponding (positive to positive and negative to negative) predictive value (expressed as odds, post-test odds). Thus, a PLR of 10 will multiply the prevalence odds in the group with a positive test by a factor of 10. NLR works in the same way. To compare different likelihood ratios of the same type, positive or negative, they are divided by each other and the quota will be the difference expressed "x-times better". For example, a PLR value of 20 is two-times better than a PLR value of 10, because 20/10 = 2. However, the NLR needs to be inverted (1/NLR) in order to enable a comparison of a PLR with a NLR, making it hard to directly access the difference between many PLR and NLR values.

**Figure 2:**
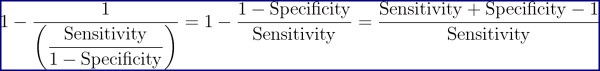
**Calculating Positive Likelihood Percentage (PLP) using sensitivity and specificity**. Inserting the formula for calculating the positive likelihood ratio using sensitivity and specificity into the newly formed formula for calculating PLP. Then the formula is simplified in two steps.

**Figure 3:**
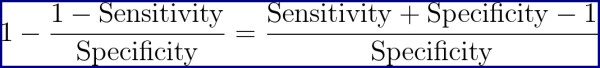
**Calculating Negative Likelihood Percentage (NLP) using sensitivity and specificity**. Inserting the formula for calculating the negative likelihood ratio using sensitivity and specificity into the newly formed formula for calculating NLP. Then the formula is simplified in one step.

In order to enable an easier comparison between different likelihood ratios, we developed an alternative complementary way to express these figures. Because positive and negative likelihood ratios are inversely related to each other, the first step in calculating likelihood percentages is to invert the positive likelihood ratio (1/PLR). All "normal" (PLR > 1, NLR < 1) likelihood ratios are now in the range between 0–1, thus they can be presented in the form of a numerical percent. However, after the first step a clear statistical result leads to a low negative and inverted positive likelihood ratio; in other statistical percentages (sensitivity, specificity, predictive values and accuracy) a clear result leads to a high percentage. In order to make a clear result lead to a high likelihood percentage the second and final step is to subtract 1/PLR or NLR from 1. Thus, the formulas are Negative Likelihood Percentage (NLP) = 1-NLR and Positive Likelihood Percentage (PLP) = 1-(1/PLR). Using the formula for calculating PLR, PLP is calculated as shown in Figure 2. Thus, PLP = (Sensitivity+Specificity-1)/Sensitivity. Using the formula for calculating NLR, NLP is calculated as shown in Figure 3. Thus, NLP = (Sensitivity+Specificity-1)/Specificity. As seen in Table 4, the classical cut-off values PLR > 10 and NLR < 0.1 are equal to PLP > 90% and NLP > 90%, respectively.

**Table 4:** Some translated values between likelihood ratios and percentages

PLR	PLP	NLR	NLP
0.5	-100%	2	-100%
1	0%	1	0%
2	50%	0.5	50%
3	67%	0.3	70%
5	80%	0.2	80%
7	86%	0.15	85%
10	90%	0.1	90%
15	93%	0.075	93%
20	95%	0.05	95%
30	97%	0.03	97%
50	98%	0.02	98%
100	99%	0.01	99%

A display of different likelihood ratios converted to likelihood percentages. Note that the positive and negative sides do not always have corresponding values on the same row.

Likelihood percentages allow fast comparison between different ratios. An example: Who would notice that the NLR value of 50–99% carotid stenoses was somewhat better than the PLR value of 70–99% stenoses without the likelihood percentage values (Table 3)? A precise comparison is made by dividing the two likelihood percentage values after they are subtracted from 1 (the percentage points that are needed to reach 100%) (1–0.62)/(1–0.64) = 1.06-times better (Note that the lesser likelihood percentage is the numerator).

There are two drawbacks with likelihood percentages: First, it is not possible to recalculate the prevalence into predictive values using likelihood percentages in the same way that it is possible using likelihood ratios without recalculating the likelihood percentages back into ratios. Second, the calculation from ratio to percentage only works for normal likelihood ratio values (PLR > 1 and NLR < 1), otherwise the percentage value becomes negative. This is seen clearly in the occlusion group of carotid stenoses (Table 3). But the intention with this new system is not to replace likelihood ratios but to be a complement where the problems with likelihood ratios lay, that is, the comparison between different ratios, especially between positive and negative ratios. Likelihood ratios and percentages are equal when used as cut-off values; perhaps likelihood percentages are slightly better because the positive and negative percentage values have the same cut-off value at 90%. Because PLP and NLP make the assessment of the difference between different likelihood ratios easier it might become a good and useful complement in the presentation of diagnostic statistics.

## Competing interests

The authors declare that they have no competing interests.

## Authors' contributions

EPJ came up with the main design of the study, co-designed the details of the study, acquired all data, analyzed all data, invented the likelihood percentage system and wrote most of the manuscript. PW came up with the idea to the study, co-designed the details of the study, wrote a part of the manuscript but most importantly gave good feed-back to EPJ about where the emphasis should lie in the manuscript. The relationship between the authors was supervisor (PW) and student (EPJ). Both authors read and approved the final manuscript.

## Pre-publication history

The pre-publication history for this paper can be accessed here:



## References

[B1] Socialstyrelsen (2005). Nationella riktlinjer för strokesjukvård 2005.

[B2] Shorr RI, Johnson KC, Wan JY, Sutton-Tyrrell K, Pahor M, Bailey JE, Applegate WB (1998). The prognostic significance of asymptomatic carotid bruits in the elderly. J Gen Intern Med.

[B3] Gillett M, Davis WA, Jackson D, Bruce DG, Davis TM (2003). Prospective evaluation of carotid bruit as a predictor of first stroke in type 2 diabetes: the Fremantle Diabetes Study. Stroke.

[B4] Mackey AE, Abrahamowicz M, Langlois Y, Battista R, Simard D, Bourque F, Leclerc J, Cote R (1997). Outcome of asymptomatic patients with carotid disease. Asymptomatic Cervical Bruit Study Group. Neurology.

[B5] Halliday A, Mansfield A, Marro J, Peto C, Peto R, Potter J, Thomas D (2004). Prevention of disabling and fatal strokes by successful carotid endarterectomy in patients without recent neurological symptoms: randomised controlled trial. Lancet.

[B6] Chambers BR, Donnan GA (2005). Carotid endarterectomy for asymptomatic carotid stenosis. Cochrane Database Syst Rev.

[B7] Yin D, Carpenter JP (1998). Cost-effectiveness of screening for asymptomatic carotid stenosis. J Vasc Surg.

[B8] Wolff T, Guirguis-Blake J, Miller T, Gillespie M, Harris R (2007). Screening for carotid artery stenosis: an update of the evidence for the U.S. Preventive Services Task Force. Ann Intern Med.

[B9] Ghilardi G (1994). [The significance of a carotid bruit. The preliminary experience of the OPI program. The Obiettivo prevenzione ictus (Stroke Prevention Objective)]. Minerva Cardioangiol.

[B10] Floriani M, Giulini SM, Bonardelli S, Portolani N, Benvenuti M, Pouche A, Tiberio G (1988). Value and limits of "critical auscultation" of neck bruits. Angiology.

[B11] Sauve JS, Thorpe KE, Sackett DL, Taylor W, Barnett HJ, Haynes RB, Fox AJ (1994). Can bruits distinguish high-grade from moderate symptomatic carotid stenosis? The North American Symptomatic Carotid Endarterectomy Trial. Ann Intern Med.

[B12] Magyar MT, Nam EM, Csiba L, Ritter MA, Ringelstein EB, Droste DW (2002). Carotid artery auscultation--anachronism or useful screening procedure?. Neurol Res.

[B13] Goldman K LR Clinical Trials no. NCT00417586. NCT00417586.

[B14] Qureshi AI, Alexandrov AV, Tegeler CH, Hobson RW, Dennis Baker J, Hopkins LN (2007). Guidelines for screening of extracranial carotid artery disease: a statement for healthcare professionals from the multidisciplinary practice guidelines committee of the American Society of Neuroimaging; cosponsored by the Society of Vascular and Interventional Neurology. J Neuroimaging.

[B15] Swedish Bureau of Statistics. http://www.scb.se.

[B16] Hansen F, Bergqvist D, Lindblad B, Lindh M, Matzsch T, Lanne T (1996). Accuracy of duplex sonography before carotid endarterectomy--a comparison with angiography. Eur J Vasc Endovasc Surg.

[B17] Ågren-Wilsson A, Backman C, Fagerlund M, Malm J (2000). [Ultrasound before carotid surgery needs to be validated locally]. Lakartidningen.

[B18] Hill SL, Holtzman G, Martin D, Evans P, Toler W (2000). Severe carotid arterial disease: a diagnostic enigma. Am Surg.

[B19] Rothwell PM, Eliasziw M, Gutnikov SA, Warlow CP, Barnett HJ (2004). Endarterectomy for symptomatic carotid stenosis in relation to clinical subgroups and timing of surgery. Lancet.

[B20] Deeks JJ, Altman DG (2004). Diagnostic tests 4: likelihood ratios. Bmj.

